# First detection, clinical presentation and phylogenetic characterization of *Porcine epidemic diarrhea virus* in Austria

**DOI:** 10.1186/s12917-015-0624-1

**Published:** 2015-12-30

**Authors:** Adolf Steinrigl, Sandra Revilla Fernández, Friedrich Stoiber, Jutta Pikalo, Tatjana Sattler, Friedrich Schmoll

**Affiliations:** Austrian Agency for Health and Food Safety, Institute for Veterinary Disease Control, Robert Koch Gasse 17, 2340 Mödling, Austria; Veterinary practice, Römerstraße 56, 4600 Wels, Austria; University of Leipzig, Large Animal Clinic for Internal Medicine, An den Tierkliniken 11, 04103 Leipzig, Germany

**Keywords:** Porcine epidemic diarrhea virus, Austria, First case, RT-qPCR, Serology

## Abstract

**Background:**

Porcine epidemic diarrhea (PED) is a syndrome that is characterized by rapidly spreading watery diarrhea affecting pigs of all ages, but with major effects on suckling piglets. The disease, as well as the causative *Alphacoronavirus*, the *Porcine epidemic diarrhea virus* (PEDV), was first described in Europe in the 1970s and since then has spread over many Asian and American countries, where it recently led to devastating effects on swine health and pork industry. While the disease was seldom reported in Europe within the last few decades, a few recent reports re-emergence of PED in German pig farms. The hitherto isolated German strain seems to be closely related to a low pathogenic PEDV variant from the USA. This case report describes the first detection of PEDV in Austria.

**Case presentation:**

Reduced feed uptake and occasional diarrhea were observed in December 2014 in a group of fattening pigs, kept on an Austrian swine farm. The concerned pigs had been recently purchased from Germany. Within a few weeks, diarrhea became apparent also in pigs of Austrian origin, which were kept in a different stable on the same farm. Gastrointestinal symptoms among fattening pigs were generally mild, quickly resolving and did not lead to death. PEDV RNA was identified by RT-qPCR in pooled feces and serum and PEDV antibodies were detectable in serum in both groups of pigs. Phylogenetic analysis of the nearly complete PEDV spike gene shows that the Austrian PEDV strain is highly similar to other strains involved in recent outbreaks in Western and Central Europe.

**Conclusion:**

This is the first report demonstrating the presence of PEDV in Austria. The virus was probably introduced by purchasing piglets from a German source, which underlines the significance of trans-boundary animal trade for the distribution of highly contagious diseases, such as PED.

**Electronic supplementary material:**

The online version of this article (doi:10.1186/s12917-015-0624-1) contains supplementary material, which is available to authorized users.

## Background

Porcine epidemic diarrhea virus (PEDV), a member of the *Coronaviridae* family, genus *Alphacoronavirus*, was first identified as the cause of porcine epidemic diarrhea (PED) in European pigs in 1978 [1]. Thereafter, PED emerged in many European countries and in Asia. While PED was seldom reported in Europe within the last two decades [2], it became a leading cause of piglet diarrhea in Asia [3]. Variant PEDV strains of high virulence emerged in China in 2010 – 2011 [4]. In 2013, PEDV that was genetically close to these highly pathogenic Chinese strains was detected in the USA for the first time, where it had an immense impact on pig health and pork business [5]. Severe vomiting and diarrhea affected pigs of all ages and mortality in suckling pigs was 90 - 95 % [6]. The disease rapidly spread from the USA to neighboring countries like Canada and Mexico and also further to the Caribbean and South America [7–9]. Contaminated transport vehicles, as well as contaminated feed, were suspected as potential sources of long-distance PEDV spread [10, 11]. Meanwhile, PEDV is present in the USA in at least three different genomic variants, which differ in pathogenicity [12–14]. The consequences on pig health and pork industry of a potential introduction of either of these PEDV strains to Europe are currently unknown. Recently, sporadic outbreaks caused by USA-like PEDV strains have been reported in Central Europe: in spring to summer 2014, PEDV was detected in several German fattening farms located in the southern and north-western parts of the country, respectively [15, 16]. Clinical symptoms were relatively mild and deaths were absent or occurred only in a small proportion of pigs. In autumn 2014, PED outbreaks occurred in two sow farms in Southern Germany, affecting both sows and piglets of different ages. Mortality in piglets was below 10 % in one sow farm, but reached up to 68 % in the other [17]. PEDV was also detected in pig farms in Belgium and France [18, 19]. Full genome sequencing revealed that all PEDV sequences isolated from recent European outbreaks are almost identical and closely related to PEDV variant strains from USA (the so-called S-INDEL strains) [14, 15, 17–19].

Here, we report the first detection of PEDV in pigs on an Austrian fattening farm and provide information about disease symptoms, diagnostic test results, possible route of PEDV entry and genetic relatedness of the newly detected Austrian PEDV to published sequences.

## Case presentation

In mid-December 2014, reduced feed uptake and mild diarrhea were observed in a pig farm located in Upper Austria in about 10–20 % of 550 fattening pigs (about 11–12 weeks of age) that had been purchased from a German source a few days before. Two pigs had died and were diagnosed with acute *Actinobacillus pleuropneumoniae* (APP) infection by pathomorphological examination and bacterial isolation, performed in a private diagnostic laboratory. Thus, antibiotic treatment of the German-origin group was initiated: pigs were treated via feed with 16 mg/kg/day Tilmicosin (Tilmovet, Virbac, Austria) for 8 days. In addition to the pigs of German origin, about 1000 fatteners of Austrian origin (aged 11–28 weeks, fresh piglets being introduced every 2–3 weeks) were housed in a different stable on the same farm. The pigs of Austrian origin were clinically inconspicuous at that time. Change of clothing between different stables was regularly practiced. Although gastrointestinal symptoms in the German-origin group were mild, the veterinary practitioner submitted a pooled feces sample for PCR testing for porcine coronaviruses, *Brachyspira* spp. and *Lawsonia intracellularis* as well as for bacterial culture and parasitological examination. Commercial reverse transcriptase quantitative PCR (RT-qPCR) kits were used for porcine coronavirus testing (EZ-PED/TGE MPX 1.0 Real-Time RT-PCR and EZ-SDCV MPX 1.0 Real Time PCR, Tetracore, USA). RT-qPCR testing revealed a high PEDV load (quantification cycle (Cq)-value of 17) in pooled feces, while tests for *Transmissible gastroenteritis virus* and *Porcine deltacoronavirus* were negative. In addition, *Brachyspira intermedia/innocens* was identified by conventional gel-based PCR (ADIAVET® BRACHY, bioMerieux, France), while the sample tested negative by *Lawsonia intracellularis* real-time PCR [20]. *Salmonella enterica* serovar Typhimurium was isolated following pre-enrichment in buffered peptone water and typed by standard methods (not shown). No endoparasites were detected in pooled feces.

By early January 2015, four weeks after the introduction of the German-origin pigs, the clinical situation in the German-origin pigs had improved and feed uptake and daily weight gain were back to normal levels. However, gastrointestinal symptoms were now apparent in >80 % of the Austrian-origin pigs, typically shown by strongly reduced feed uptake and diarrhea that lasted for 2–3 days. No vomiting was observed and no deaths occurred. Pooled feces collected from both German- and Austrian-origin animals (each ten pen-wise pooled samples) tested positive for PEDV in RT-qPCR (Cq-values ranging from 15–41), with lowest Cq-values (indicative of highest PEDV-load) in samples originating from pigs that had been introduced just a few days ago. These animals were diarrheic at the time of sampling. The mean PEDV Cq-value was lower in feces from the Austrian-origin pigs than from the German-origin pigs (23 Cq vs. 31 Cq). *Brachyspira* spp. was identified by PCR in a fraction of samples from both groups. *Oesophagostomum* sp. was demonstrated in a single pooled feces sample from the Austrian-origin pigs, while *Salmonella* Typhimurium was again identified in feces from the German-origin pigs, but not from those of Austrian origin.

PEDV was further demonstrated by RT-qPCR in 40–50 % of sera collected from both German- and Austrian-origin pigs (each ten samples), respectively. PEDV Cq-values in sera were relatively high (> Cq 31) in both groups. PEDV-specific antibodies (PEDV-Ab) were detectable by commercial ELISA (Swinecheck® PED indirect; Biovet, Canada) in all sera from the German-origin group and five from the Austrian-origin group; the Austrian-origin pigs that showed diarrhea (pens A7 - A10) were PEDV-Ab negative (Additional file 1: Table S1).

Eleven days later (~ mid-January 2015), clinical symptoms were no longer evident in any of the groups. Nevertheless, 70–91 % of pooled feces samples from both groups still tested positive by PEDV RT-qPCR. In comparison to the previous sampling time-point (early January), mean Cq-values in feces had decreased in both groups, ranging now from Cq 32 - Cq 34, which indicates a drop in PEDV fecal shedding. Furthermore, PEDV-Ab were now detectable in sera collected from those pens that had shown PEDV-Ab negative results 11 days before. Sera from two pens were still positive for PEDV RNA at low level (Additional file 1: Table S1). Pigs that were freshly purchased around the third sampling time-point and housed with the Austrian-origin group no longer became infected as indicated by absence of gastrointestinal symptoms and lack of seroconversion. Nevertheless, by RT-qPCR, PEDV remained detectable in regularly submitted pooled feces until early March 2015, albeit in declining frequency.

During and after these follow-up studies on the index farm, PED was confirmed in our laboratory on three additional pig finishing farms located in Upper Austria in February - June 2015. In addition, PEDV was detected in an intestinal sample collected from a finishing farm in neighboring Slovenia in February 2015. Veterinarians reported highly varying degrees of mortality (7–100 %), but absent or low (<1 %) mortality. All affected farms were specialized on finishing pigs. Permission for taking samples was granted by all farm owners.

## Molecular epidemiological investigation

**Fig. 1:**
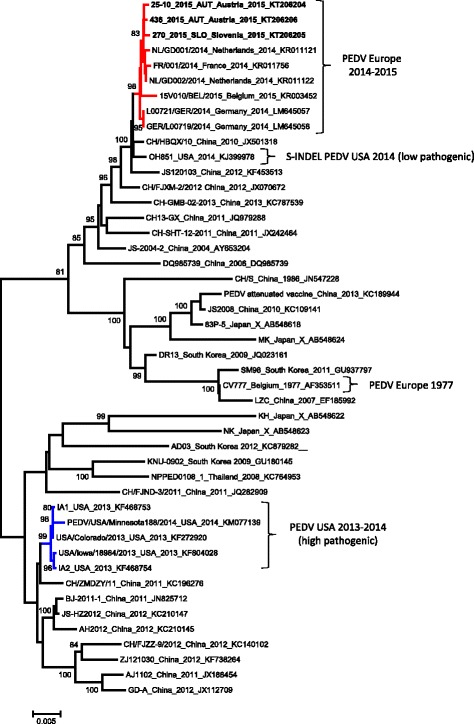
Neighbor-joining phylogenetic tree (Maximum Likelihood Composite substitution model) based on a 4091 bp alignment (gaps excluded) of the nearly complete PEDV spike protein (PEDVgp2) coding region from 43 PEDV sequences downloaded from GenBank and from three PEDV newly reported in this paper (GenBank accession numbers: KT206204 - KT206206). Multiple sequence alignment was performed using the ClustalW algorithm, as implemented in BioEdit v7.1.3.0 [25]. Numbers along the branches indicate the percentage of 1000 bootstrap iterations (bootstrap support < 80 % is not shown). For each sequence, the strain designation, followed by country and year (if known, otherwise this is indicated by X) of isolation and the GenBank accession number is shown. PEDV strains from Europe and the USA are indicated by brackets and partly by colored branches. PEDV sequences from Austria and Slovenia are indicated in bold

To determine the genetic relationship of PEDV detected on Austrian and Slovenian pig farms to hitherto published strains, sequencing of the PEDV S-gene from selected RT-qPCR positive samples was conducted. Briefly, the complete S-gene was amplified by RT-PCR, utilizing primers S-F1 and S-R1 [21], followed by direct sequencing of the RT-PCR product using a series of nine sequencing primers (Additional file 2: Table S2). A phylogenetic tree was constructed based on an alignment of the nearly complete S-gene (4091 sites, gaps excluded), using MEGA5 software [22]. As shown in Fig. 1, PEDV detected in Austria and Slovenia belongs to a highly supported lineage (98 % bootstrap value) including also recent PEDV sequences from Belgium, Germany, France and the Netherlands [15, 18, 19]. Within the S-gene, nucleotide sequence identity between these sequences is > 99.5 %.

## Discussion

In this report, the first detection of PEDV on an Austrian pig fattening farm is described. Clinical symptoms, such as decreased feed uptake, reduced weight gain and diarrhea initially affected pigs that had been introduced from Germany a few days before. Within a few weeks, this condition had already spread to pigs of Austrian origin that were kept in a different stable on the same farm. PEDV was demonstrated by RT-qPCR in both feces and serum of affected pigs. RT-qPCR results in feces were further confirmed by conventional PCR and sequencing [21, 23]. Furthermore, presence of *Brachyspira* spp., *Oesophagostomum* sp. and *Salmonella* Typhimurium was demonstrated in pooled feces. The chronological appearance of clinical symptoms in the two different groups of pigs was in accordance with semi-quantitative RT-qPCR results (Cq-values) in pen-wise pooled feces and with the order of occurrence of PEDV-Ab in serum. PEDV RNA was detected in sera from both diarrheic and convalescent pigs, although in the latter at very low level. Presence of PEDV RNA in serum is consistent with the acute and later phases of infection, as already described by others [24].

A similar onset of disease following introduction of feeder pigs and rapid spread within the farms was reported from several recent outbreaks in German fattening farms [16, 17]. In comparison to other reported outbreaks in fattening pigs [16–18], the PED case report presented here was characterized by mild enteric disease that resolved within 2–3 days; furthermore, no pigs died from diarrhea or dehydration. The death of two pigs at the beginning of the outbreak was due to APP infection. Interestingly, PEDV was detectable on the Austrian farm by RT-qPCR for an extended period of time (almost 3 months) in pooled feces, although clinical symptoms on the farm had completely resolved 4–5 weeks after presumptive introduction of PEDV. From that time onwards, no freshly introduced seronegative pigs became infected, although they were housed in the same stable with previously affected pigs. This might indicate that PEDV detected by RT-qPCR after cessation of clinical signs was no longer infectious.

High morbidity, but low or no mortality among fattening pigs was also observed during a PED epidemic in 2005–2006 in Italy [2]. In contrast, highly varying degrees of mortality (6–68 %) among suckling and weaned piglets were observed on two German sow farms and on a French farrow-to-finish herd [17, 18]. While the sequence of the virus responsible for the Italian outbreak in 2005–2006 has not been reported yet, full genome sequencing of recent PEDV strains from Belgium, France and Germany shows that they are all highly similar and closely related to S-INDEL strains from USA, such as OH851 [15, 17]. Phylogenetic analysis based on the S-gene sequence shows that the virus recovered from PED outbreaks in Austria and neighboring Slovenia is almost identical to other currently circulating PEDV from Western and Central Europe [15, 18, 19], which suggests a common source of introduction.

## Conclusions

This case report describes the first detection and characterization of PEDV on an Austrian pig farm. PEDV was introduced most likely by purchasing piglets from a German source. The infection spread quickly between different stables of the same farm and led to mild disease in fattening pigs without any deaths related to enteric disease. PEDV RNA was detected in pooled feces for an extended period of time after cessation of clinical symptoms and in the absence of new infections. So far, all available sequences point to a single PEDV S-INDEL strain as the cause of recent PED outbreaks in Western and Central Europe that are, nevertheless, characterized by varying disease severity.

## Ethics statement

All diagnostic and therapeutic procedures involving live animals were performed by an approved veterinarian in the course of routine veterinary health management.

## Availability of supporting data

Supporting data (Additional file 1: Table S1 and Additional file 2: Table S2) are included as additional files to this article. Sequence data are deposited under GenBank accession numbers KT206204 - KT206206.

## Additional files

Additional file 1: Table S1. PEDV RT-qPCR and ELISA results obtained from serum samples in January 2015. (DOCX 20 kb) Additional file 2: Table S2. Primers used for amplification and sequencing of the complete S-gene. (DOCX 20 kb)

## Abbreviations

PED: Porcine epidemic diarrhea
PEDV: Porcine epidemic diarrhea virus
RNA: Ribonucleic acid
PCR: Polymerase chain reaction
RT-qPCR: Reverse transcriptase quantitative PCR
APP: *Actinobacillus pleuropneumoniae*
Cq: Quantification cycle
ELISA: Enzyme linked immunosorbent assay
PEDV-Ab: Anti-PEDV antibodies
S-INDEL strain: PEDV strain having insertions and deletions in the spike gene
