# Erratum to: First detection, clinical presentation and phylogenetic characterization of *Porcine epidemic diarrhea virus* in Austria

**DOI:** 10.1186/s12917-016-0647-2

**Published:** 2016-02-01

**Authors:** Adolf Steinrigl, Sandra Revilla Fernández, Friedrich Stoiber, Jutta Pikalo, Tatjana Sattler, Friedrich Schmoll

**Affiliations:** Austrian Agency for Health and Food Safety, Institute for Veterinary Disease Control, Robert Koch Gasse 17, 2340 Mödling, Austria; Veterinary practice, Römerstraße 56, 4600 Wels, Austria; University of Leipzig, Large Animal Clinic for Internal Medicine, An den Tierkliniken 11, 04103 Leipzig, Germany

Unfortunately, after publication of this manuscript [1], it was found that the Case Presentation section contained an error. The line that reads, “Veterinarians reported highly varying degrees of mortality (7–100 %), but absent or low (<1 %) mortality” should instead read, “Veterinarians reported highly varying degrees of morbidity (7–100 %), but absent or low (<1 %) mortality.”
