# Supplementary material for: First detection, clinical presentation and phylogenetic characterization of Porcine epidemic diarrhea virus in Austria
Source: BMC Vet Res. 2015 Dec 30;11:310. doi: 10.1186/s12917-015-0624-1 (PMC4696200; doi:10.1186/s12917-015-0624-1)
Supplement: Additional file 1: Table S1. — PEDV RT-qPCR and ELISA results obtained from serum samples in January 2015. (DOCX 20 kb) [file 12917_2015_624_MOESM1_ESM.docx]

Supplemental table 1

PEDV RT-qPCR and ELISA results obtained from serum samples in January 2015

| **Sampling date** | **Pen-ID*** | **PEDV RT-qPCR** | | **PEDV ELISA** | |
| --- | --- | --- | --- | --- | --- |
|  |  | **qualitative** | **Cq-value** | **qualitative** | **S/P value** |
| 09.01.2015 | A1 | neg | No Cq | **pos** | **1.081** |
|  | A2 | neg | No Cq | **pos** | **2.739** |
|  | A3 | neg | No Cq | **pos** | **2.679** |
|  | A4 | neg | No Cq | **pos** | **3.167** |
|  | A5 | neg | No Cq | **pos** | **2.827** |
|  | A6 | **pos** | **37.56** | neg | 0.307 |
|  | A7 | **pos** | **32.64** | neg | 0.091 |
|  | A8 | **pos** | **31.28** | neg | 0.000 |
|  | A9 | **pos** | **31.61** | neg | 0.023 |
|  | A10 | **pos** | **39.59** | neg | 0.010 |
|  | G1 | **pos** | **35.66** | **pos** | **1.444** |
|  | G2 | neg | No Cq | **pos** | **1.655** |
|  | G3 | neg | No Cq | **pos** | **0.958** |
|  | G4 | neg | No Cq | **pos** | **0.549** |
|  | G5 | **pos** | **35.76** | **pos** | **1.009** |
|  | G6 | **pos** | **35.95** | **pos** | **1.463** |
|  | G7 | **pos** | **35.55** | **pos** | **0.462** |
|  | G8 | neg | No Cq | **pos** | **0.950** |
|  | G9 | neg | No Cq | **pos** | **1.562** |
|  | G10 | neg | No Cq | **pos** | **1.534** |
| 20.01.2015 | A6 | neg | No Cq | **pos** | **2.226** |
|  |  | neg | No Cq | **pos** | **3.573** |
|  |  | neg | No Cq | **pos** | **3.161** |
|  | A7 | neg | No Cq | **pos** | **3.717** |
|  |  | neg | No Cq | **pos** | **3.237** |
|  |  | neg | No Cq | **pos** | **1.480** |
|  | A8 | neg | No Cq | **pos** | **2.476** |
|  |  | neg | No Cq | **pos** | **2.218** |
|  |  | neg | No Cq | **pos** | **0.800** |
|  | A9 | neg | No Cq | **pos** | **1.650** |
|  |  | neg | No Cq | **pos** | **3.252** |
|  |  | **pos** | **36.1** | **pos** | **3.228** |
|  | A10 | neg | No Cq | **pos** | **0.779** |
|  |  | **pos** | **37.3** | **pos** | **1.546** |
|  |  | **pos** | **43.3** | **pos** | **2.939** |

*Pens with A contained Austrian origin pigs, whereas pens with G contained German origin pigs. Positive results are indicated by grey shading. The PEDV ELISA cut-off is 0.4.
