# Supplementary material for: First detection, clinical presentation and phylogenetic characterization of Porcine epidemic diarrhea virus in Austria
Source: BMC Vet Res. 2015 Dec 30;11:310. doi: 10.1186/s12917-015-0624-1 (PMC4696200; doi:10.1186/s12917-015-0624-1)
Supplement: Additional file 2: Table S2. — Primers used for amplification and sequencing of the complete S-gene. (DOCX 20 kb) [file 12917_2015_624_MOESM2_ESM.docx]

Supplemental table 2

Primers used for amplification and sequencing of the complete S-gene

| **Primer name** | **Sequence 5‘-3‘** | **Purpose** | **Position*** | **Reference** |
| --- | --- | --- | --- | --- |
| S-F1 | TGCTAGTGCGTAATAATGAC | Amplification and sequencing | 20573 - 20592 | [[1](#_ENREF_1)] |
| S-R1 | CATCTTTGACAACTGTGT | Amplification | 24833 - 24816 | [[1](#_ENREF_1)] |
| PEDV_267_F | TCTGGTCAGGGCTTTGAGAT | Sequencing | 20880 - 20899 | This study |
| PEDV_796_F | TTTGTCCAATGATTCCACTTTG | Sequencing | 21407 - 21428 | This study |
| PEDV_1296_F | CATGGCACTGACGATGATGT | Sequencing | 21909 - 21928 | This study |
| PEDV_1777_F | GTGTTTCCACCAGCCTTTTG | Sequencing | 22390 - 22409 | This study |
| PEDV_2295_F | ATTGGCTACGTCCCATCTCA | Sequencing | 22908 - 22927 | This study |
| PEDV_2768_F | GCTGTTCTAATGGTCGCTCTG | Sequencing | 23380 - 23400 | This study |
| PEDV_3274_F | CCGGCAGATTATCAGCACTT | Sequencing | 23887 - 23906 | This study |
| PEDV_3788_F | GCCCAATAGAACTGGTCCAA | Sequencing | 24401 - 24420 | This study |

*Position corresponds to PEDV strain GER/L00721/2014 (GenBank accession no. LM645057)

1. Huang YW, Dickerman AW, Pineyro P, Li L, Fang L, Kiehne R, Opriessnig T, Meng XJ: **Origin, evolution, and genotyping of emergent porcine epidemic diarrhea virus strains in the United States**. *mBio* 2013, **4**(5):e00737-00713.
